# Knockdown of Circ_0037658 Alleviates IL-1β-Induced Osteoarthritis Progression by Serving as a Sponge of miR-665 to Regulate ADAMTS5

**DOI:** 10.3389/fgene.2022.886898

**Published:** 2022-08-24

**Authors:** Ningbo Li, Yongsheng Wang, Xuejian Wu

**Affiliations:** ^1^ Department of Orthopedics, The First Affiliated Hospital of Zhengzhou University, Zhengzhou, China; ^2^ Department of Orthopedics, The First Affiliated Hospital of Henan University of Chinese Medicine, Zhengzhou, China; ^3^ Department of Orthopedics, The First Affiliated Hospital of Henan University, Kaifeng, China

**Keywords:** circ_0037658, miR-665, ADAMTS5, IL-1β, osteoarthritis

## Abstract

**Background:** Osteoarthritis (OA) is a chronic musculoskeletal degeneration disease which brings great pain to patients and a tremendous burden on the world’s medical resources. Previous reports have indicated that circular RNAs (circRNAs) are involved in the pathogenesis of OA. The purpose of this study was to explore the role and mechanism of circ_0037658 in the OA cell model.

**Methods:** The content of interleukin-6 (IL-6) and tumor necrosis factor α (TNF-α) was measured using enzyme-linked immunosorbent assay (ELISA). Cell proliferation ability and apoptosis were detected using Cell Counting Kit-8 (CCK-8), 5-ethynyl-2′-deoxyuridine (EDU), and flow cytometry assays. Western blot assay was used to measure the protein levels of Bcl-2-related X protein (Bax), cleaved-caspase-3, MMP13, Aggrecan, and ADAMTS5. The expression of circ_0037658, microRNA-665 (miR-665), and a disintegrin and metalloproteinase with thrombospondin motifs (ADAMTS) 5 was detected using real-time quantitative polymerase chain reaction (RT-qPCR). Dual-luciferase reporter assay and RNA Immunoprecipitation (RIP) assay were manipulated to analyze the relationships of circ_0037658, miR-665, and ADAMTS5.

**Results:** Human chondrocytes (CHON-001 cells) were treated with interleukin-1β (IL-1β) to establish an OA cell model. Circ_0037658 and ADAMTS5 levels were increased, and miR-665 was decreased in OA cartilage samples and IL-1β-treated chondrocyte cells. Moreover, circ_0037658 silencing promoted proliferation and impaired inflammation, apoptosis, and ECM degradation in IL-1β-treated CHON-001 cells. Mechanically, circ_0037658 acted as a sponge for miR-665 to regulate ADAMTS5 expression.

**Conclusion:** Circ_0037658 knockdown relieved IL-1β-triggered chondrocyte injury via regulating the miR-665/ADAMTS5 axis, promising an underlying therapeutic strategy for OA.

## Highlights

1) IL-1β suppressed CHON-001 cell growth *in vitro*
2) Circ_0037658 knockdown overturned IL-1β-mediated CHON-001 cell growth
3) Circ_0037658 directly interacted with miR-665
4) ADAMTS51 was the target of miR-665

## Introduction

Osteoarthritis (OA) is a kind of degenerative joint disease that has become an international health issue and caused huge costs to the world society (Martel-Pelletier et al., 2016). Characteristically, synovial inflammation, articular cartilage injury, and cartilage extracellular matrix (ECM) degradation can lead to swelling, pain, and joint failure (Brandt et al., 2008; Musumeci et al., 2015). Currently, the risk factors for OA are mainly post-traumatic, age-related, obesity, joint deformities, and genetic factors (Bijlsma et al., 2011; Glyn-Jones et al., 2015). With the development of the aging world population, it is also one of the diseases with the fastest growing incidence (Liu et al., 2018). Conventional methods have made significant progress in recent years and achieved certain benefits, but the high costs impede their wide application (Mathiessen and Conaghan, 2017; Honvo et al., 2019). Of interest, previous studies have verified that chondrocytes are the only resident cells in the articular system and are critical for maintaining proper articular cartilage (Charlier et al., 2016). Moreover, as an important inflammatory factor, interleukin-1β (IL-1β) is related to the pathogenesis of OA, and its release can contribute to the production of some inflammatory mediators, which are responsible for chondrocyte dysfunction and ECM degradation (Goldring et al., 2011; Chen et al., 2018; Cook et al., 2018). In recent decades, numerous reports have exhibited that the IL-1β-modified chondrocytes can be widely applied to *in vitro* cell models of OA (Chang et al., 2012; Huang et al., 2020). Hence, in-depth exploration of the molecular mechanisms of OA cell models is urgent for developing novel strategies for OA treatment.

Over the last decade, there has been increasing evidence that most mammalian genomes are actively transcribed, producing non-coding RNAs that play different roles in various cellular processes (Hombach and Kretz, 2016). Circular RNAs (circRNAs) are a widespread form of non-coding RNAs, which have aroused great interest in the public due to the unique covalently closed loops formed by non-canonical back-splicing of pre-mRNA transcripts (Conn et al., 2015; Kristensen et al., 2019). Increasing literature has supported that circRNAs are usually tissue/stage-specific and associated with the development of various human diseases (Li et al., 2019; Wang L. et al., 2021). Dysregulation of circRNAs in OA is associated with chondrocyte growth, inflammation, and other pathological processes (Mao et al., 2021; Zhang et al., 2021). Some circRNAs (circ_DHRS3 and circ_0134111) promote IL-1β-triggered chondrocyte apoptosis and ECM degradation in a microRNA (miRNA)-mRNA dependent manner (Jiang et al., 2021; Wu et al., 2021). CircRNA.33186 knockdown alleviated IL-1β-induced chondrocyte cell injuries via sponging miR-127–5p (Zhou et al., 2019). Interestingly, the circRNA expression profile revealed that circ_0037658 in osteoarthritic synovium was a significantly upregulated circRNA (Xiang et al., 2019). In addition, circ_0037658 knockdown protected IL-1β-aroused cell injury via inducing autophagy (Sui et al., 2021). However, its precise role and molecular mechanism in the IL-1β-induced OA cell model remain to be further studied.

At present, research on the regulatory mechanism of circRNAs-miRNA-mRNA has become an important focus in RNA research (Hansen et al., 2013; Rong et al., 2017). It is pointed out that circRNAs play an essential regulator in many diseases through efficiently binding and repressing miRNA transcription, thereby affecting the downstream mRNA expression (Kulcheski et al., 2016; Panda, 2018). Herein, Starbase software analysis presented that circ_0037658 interacts with miR-665. Meanwhile, miR-665 was involved in the regulation of IL-1β-cell damage in some publications (Ouyang et al., 2021). Therefore, this study aimed to investigate the effects of circ_0037658 and miR-665 on IL-1β-induced cell injury and their relationship.

## Materials and Methods

### Clinical Tissue Collection and Cell Culture

The approval for performing the project was provided by the Ethics Committee of the First Affiliated Hospital of Zhengzhou University. Cartilage tissue specimens were collected from 21 OA patients undergoing total knee arthroplasty and 17 healthy traumatic amputees at the First Affiliated Hospital of Zhengzhou University, and the written informed consent from these subjects was obtained before enrolling.

Human chondrocyte cell line CHON-001 cells (CRL-2846, ATCC, Manassas, VA, United States) were cultured in DMEM (30–2002, ATCC) at 37°C, 5% CO_2_ with 0.1 mg/mL G-418, and 10% fetal bovine serum (FBS; PAN Biotech, Aidenbach, Germany). In addition, IL-1β (Sigma-Aldrich, St. Louis, MO, United States) at different doses (0, 5, 10, and 15 ng/ml) was used to stimulate CHON-001 cells for 24 h at 37°C to mimic the OA chondrocyte model *in vitro* as previously described (Cheleschi et al., 2018; Wang et al., 2019). 10 ng/ml IL-1β treatment was selected for further investigation.

### Enzyme-Linked Immunosorbent Assay (ELISA)

After multiple treatments, the CHON-001 cell culture medium was harvested and the levels of IL-6 and tumor necrosis factor α (TNF-α) were subsequently measured, according to the operation manual of specific ELISA kits (R&D Systems, Minneapolis, MN, United States).

### Cell Proliferation

For the Counting Kit (CCK-8) assay, un-treated or treated CHON-001 cells (approximately 2000 cells/well) were plated into 96-well plates at 37°C conditions containing 5% CO_2_. After 24 h of culture, 10 μL CCK-8 reagent (Dojindo, Kumamoto, Japan) was mixed for 4 h. At indicated time points, the determination of absorbance was conducted using a microplate reader at 450 nm. For the 5-ethynyl-2′-deoxyuridine (EdU) assay, 2×10^5^ cells were introduced into 24-well plates, followed by a mixture with 50 μM EdU (Solarbio, Beijing, China) for 2 h. After being fixed in a 4% formaldehyde solution, cells were reacted with the Apollo reaction cocktail and DAPI at 37°C in the dark, followed by visualization under a fluorescence microscope.

### Cell Apoptosis Assay

In general, CHON-001 cells were harvested and 1×10^6^ cell suspension was prepared. After being washed and digested, treated cells were re-suspended in 100 μl binding buffer and incubated with 5 μl Annexin V-FITC and 10 μl PI (Bender Med System, Vienna, Austria) at room temperature away from light. 15 min later, cell apoptosis was sorted and analyzed with the help of a flow cytometer.

### Western Blot Assay

Using a proteinase inhibitor mixture in RIPA buffer (Beyotime, Shanghai, China), lysis of tissue samples and CHON-001 cells was prepared. Subsequently, an equal amount of protein was separated by 10% SDS-PAGE and transferred onto nitrocellulose membranes (Millipore, Molsheim, France), which were then blocked with 5% non-fat milk. After hybridization with the primary antibodies: Bax (ab53154), Cleaved-caspase-3 (1:1000, ab2302), MMP13 (1:1000, ab39012), Aggrecan (1:1000, ab36861), ADAMTS5 (1:1000, ab45047), and GAPDH (1:1000, ab9485) all night at 4°C, the membranes were incubated with secondary antibody (1:10000, ab205718) for 1 h at 37°C. At last, the protein blots were subjected to the enhanced chemiluminescence (ECL) reagent (Millipore) for visualization.

### Real-Time Quantitative Polymerase Chain Reaction (RT-qPCR)

Generally, RNA extraction in clinical specimens or CHON-001 cells was accomplished using TRIzol (Invitrogen, Paisley, Scotland, United Kingdom), and their 260/280 value was distributed from 1.8 to 2.0. Furthermore, all RNAs were treated with 3 U/μg of RNase R (Epicentre, Madison, WI, United States). For circRNA and mRNA, reverse transcription was carried out according to the Bio-Rad iScript kit (Bio-Rad Laboratories, Hercules, CA, United States), followed by RT-qPCR using iQSYBR Green SuperMix (Bio-Rad). For miRNA detection, All-in-One™ miRNA First-Strand cDNA Synthesis kit and the All-in-One™ miRNA qPCR kit (GeneCopoiea, Rockville, MD, United States) were applied, referring to the operation manual. Samples were normalized to GAPDH and U6, and the 2^–ΔΔCt^ method analyzed the relative expression. The primers were listed in Table 1.

**TABLE 1:** Sequences of primers for RT-qPCR used in this study.

Names	Sequences (5′-3′)
hsa_circ_0037658: Forward	CCA​GAC​GAC​AAT​TTC​AAA​GGA
hsa_circ_0037658: Reverse	GGT​GCA​GTG​GTG​ACT​GTG​TC
ADAMTS5: Reverse	AAA​GGG​GAG​AAT​CTG​CCT​GC
ADAMTS5: Reverse	CCA​AGA​TCC​CCA​GTT​GCC​AT
miR-665: Forward	GTA​TGA​GAC​CAG​GAG​GCT​GA
miR-665: Reverse	CTC​AAC​TGG​TGT​CGT​GGA​G
U6: Forward	CTC​GCT​TCG​GCA​GCA​CAT​A
U6: Reverse	CGA​ATT​TGC​GTG​TCA​TCC​T
GAPDH: Forward	AAG​GCT​GTG​GGC​AAG​GTC​ATC
GAPDH: Reverse	GCG​TCA​AAG​GTG​GAG​GAG​TGG

### Cell Transfection

Small interfering RNAs specific to circ_0037658 (si-circ_0037658), the inhibitors and mimic of miR-665 (miR-665 and anti-miR-665), and their negative controls (si-NC, miR-NC, and anti-miR-NC) were obtained from Ribobio (Guangzhou, China). Meanwhile, overexpression vectors pCD5-circ_0037658 (circ_0037658) and ADAMTS5, and their controls (pCD5-ciR and pcDNA), were purchased from Geneseed (Guangzhou, China). Subsequently, CHON-001 cells at 60% confluence were transfected with the above transfection agents using Lipofectamine 3000 reagent (Invitrogen) for 48 h. After that, the transfection efficiency of the harvested CHON-001 cells was detected using RT-qPCR or western blot, followed by treatment with IL-1β.

### Dual-Luciferase Reporter Assay

Using Starbase software, the prediction of the binding sequences was achieved. Sequence fragment of circ_0037658 and ADAMTS53′ untranslated region (3′UTR) containing miR-665-matched binding sites or miss-matched target sites was designed and inserted into the pGLO reporter vector (Promega, Fitchburg, WI, United States) by Hanbio (Shanghai, China), generating WT/MUT-circ_0037658 and WT/MUT-ADAMTS5 3′UTR reporter vectors. Co-transfection of CHON-001 cells was conducted with the generated vectors and miR-665 or miR-NC for 48 h, followed by the analysis of luciferase activities in cell lysates was performed using a dual-luciferase reporter assay kit (Promega).

### RNA Immunoprecipitation (RIP)

In this assay, a commercial Magna RNA immunoprecipitation kit (Millipore) was applied to verify the interaction between miR-665 and circ_0037658 or ADAMTS5 in CHON-001 cells. In short, CHON-001 cells at 80% confluency were incubated with complete RIP lysis buffer. Then, cell lysates were incubated with anti-Argonaute2 (Ago2, ab186733, 1:50, Abcam) or immunoglobulin G (IgG, ab172730, 1:100, Abcam) for 4 h at 4°C before treating magnetic protein A/G beads for 2 h. After being digested with proteinase K, the RNAs on the beads were retrieved and analyzed using RT-qPCR.

### Statistical Analysis

The collected data were exhibited as mean ± standard deviation (SD) and processed according to GraphPad Prism8 software. Data differences were compared according to Student’s *t*-test or one-way analysis of variance (ANOVA) with Tukey’s tests. The expression association was assessed using Pearson correlation analysis. *p*-value < 0.05 was deemed as a statistically significant difference.

## Results

### IL-1β Induced Human Chondrocyte Damage

**FIGURE 1:**
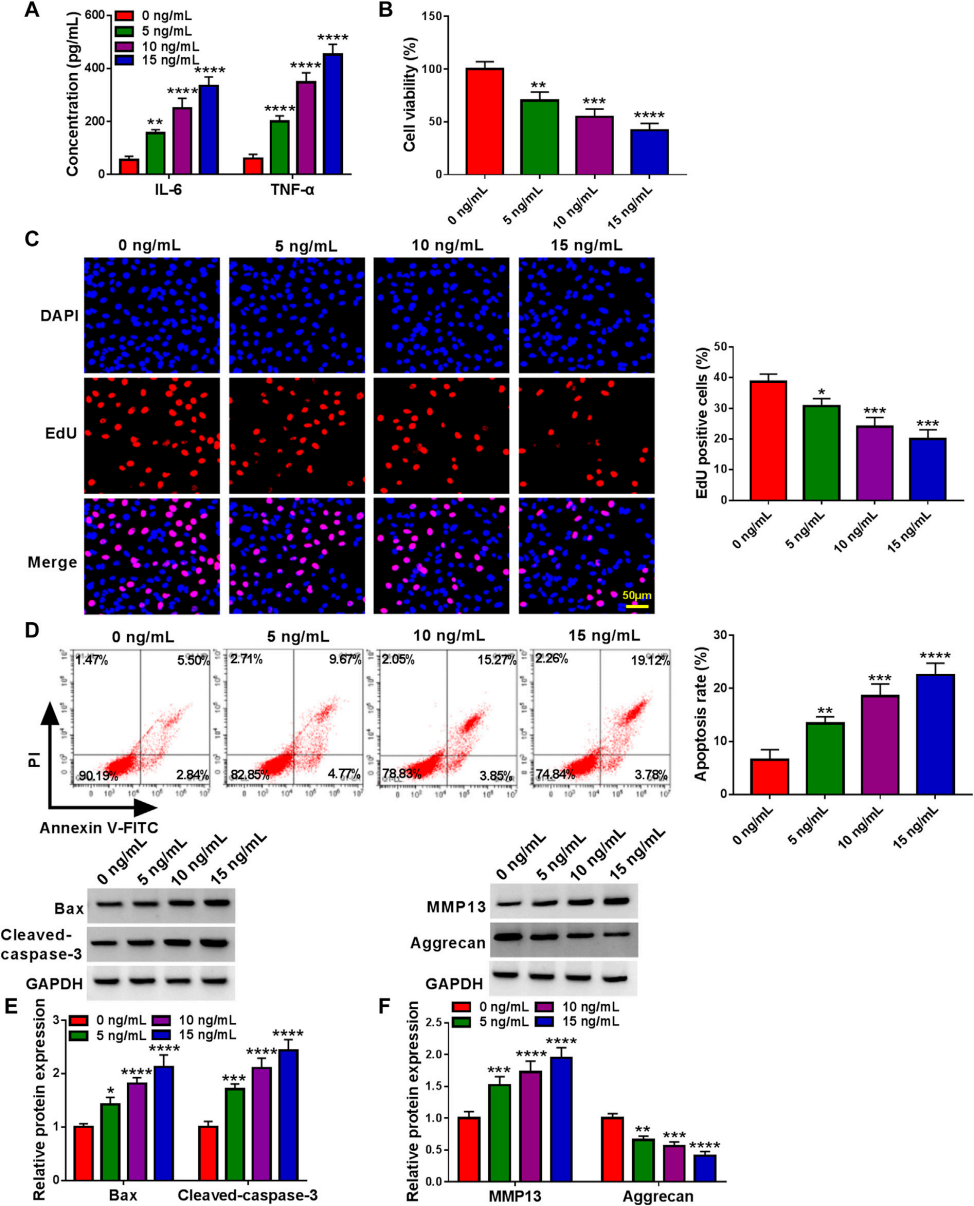
Effects of IL-1β on inflammation, proliferation, apoptosis, and ECM degradation of chondrocytes. CHON-001 cells were treated with 0, 5, 10 or 15 ng/ml IL-1β for 24 h. **(A)** Concentration of cytokines (IL-6 and TNF-α) in CHON-001 cells’ culture medium of different groups was measured by ELISA Kit. **(B)** CCK-8 assay was applied to examine cell viability in treated CHON-001 cells. **(C)** EdU assay was carried out to detect proliferative cells in treated CHON-001 cells. **(D)** Flow cytometry assay was performed to determine apoptosis rate in treated CHON-001 cells. **(E,F)** Bax, Cleaved-caspase-3, MMP13, and Aggrecan were measured using western blot assay in treated CHON-001 cells, and GAPDH was used as a loading control. These results were presented as the mean ± (SD), n = 3. *, **, ***or **** indicates *p* < 0.05, *p* < 0.01, *p* < 0.001, or *p* < 0.0001 relative to the 0 ng/ml IL-1β groups.

An *in vitro* model of OA was established in human articular chondrocyte (CHON-001 cells) treated with IL-1β. Data displayed that the secretions of pro-inflammatory cytokines in cells were highly induced in a dose-dependent manner upon IL-1β (Figure 1A). Subsequently, cell proliferation ability gradually declined after treatment of 0–15 ng/ml IL-1β for 24 h (Figures 1B,C). On the contrary, the cell apoptosis rate was significantly elevated in IL-1β-administered chondrocytes (Figure 1D). Consistently, the western blot assay presented a significant enhancement in Bax and Cleaved-caspase-3 of treated cells (Figure 1E). Furthermore, previous studies have confirmed that MMP13 upregulation (a key cartilage-degrading enzyme) and Aggrecan downregulation (a main proteoglycan in the articular cartilage) are critical events in the early stage of OA (Burrage et al., 2006; Troeberg and Nagase, 2012; Haller et al., 2015). As expected, the western blot assay verified that the treatment of IL-1β led to a significant increase in MMP13 and a substantial decrease in Aggrecan in CHON-001 cells (Figure 1F). Together, IL-1β treatment might induce cell injury in OA, and 10 ng/ml IL-1β treatment for 24 h was chosen to mimic chondrocyte injury for further research.

### Circ_0037658 Knockdown Recovered the Effects on Inflammation, Proliferation, Apoptosis, and ECM Degradation in IL-1β-Administered Chondrocytes

**FIGURE 2:**
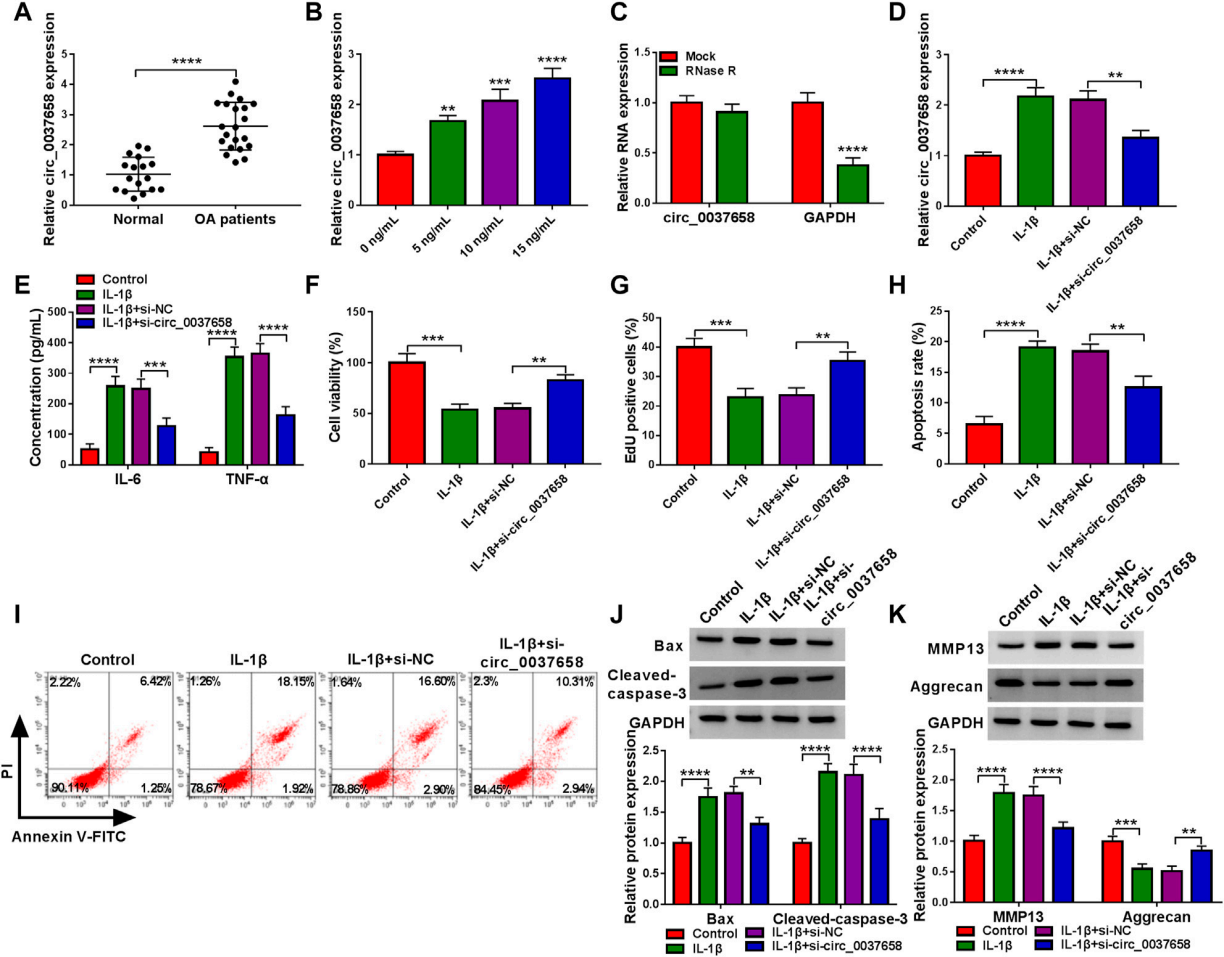
IL-1β-triggered chondrocyte injury was abolished by circ_0037658 silencing. **(A)** RT-qPCR analysis of circ_0037658 expression level in 21 OA cartilage tissues and 17 healthy cartilage tissues (**** indicates *p* < 0.0001, relative to the control group). **(B)** Relative circ_0037658 expression in CHON-001 cells was detected using RT-qPCR assay with different doses of IL-1β (**, ***or **** indicates *p* < 0.01, *p* < 0.001, or *p* < 0.0001 relative to the 0 ng/ml IL-1β groups). **(C)** RT-qPCR analysis of circ_0037658 expression after treatment with RNase R in CHON-001 cells. **(D–K)** CHON-001 cells were treated with IL-1β, IL-1β+si-NC, IL-1β+si-circ_0037658, or without (Control) (**** indicates *p* < 0.0001 relative to the Mock group). **(D)** The expression level of circ_0037658 was gauged in treated CHON-001 cells using RT-qPCR assay. **(E)** The concentration of IL-6 and TNF-α in treated CHON-001 cells was analyzed using ELISA Kit. **(F,G)** CHON-001 cell proliferative ability was determined using CCK-8 assay and EdU assay. **(H,I)** CHON-001 cell apoptosis rate in different groups was monitored using flow cytometry assay. **(J,K)** Western blot analysis of Bax, Cleaved-caspase-3, MMP13, and Aggrecan protein levels in treated CHON-001 cells, and GAPDH was used as a loading control. **(D–K)**: *** or **** indicates *p* < 0.001 or *p* < 0.0001 when IL-1β group was compared to the negative control group and **, ***, or **** indicates *p* < 0.01, *p* < 0.001 or *p* < 0.0001 when IL-1β+si-circ_0037658 group was compared to the IL-1β+si-NC group). These results were presented as the mean ± (SD), n = 3. **p < 0.01, ***p < 0.001, ****p < 0.0001.

According to the data shown in Figure 2A, the circ_0037658 level was significantly increased in 21 OA cartilage tissues versus 17 healthy cartilage tissues using the RT-qPCR assay. Consistently, our data verified that circ_0037658 was highly expressed in IL-1β-stimulated CHON-001 cells in a dose-dependent manner (Figure 2B). Whereafter, the circ_0037658 was resistant to RNase R in CHON-001 cells, and linear GAPDH was reduced (Figure 2C). Subsequently, the transfection efficiency of si-circ_0037658 was verified in Figure 2D. After that, the results of ELISA presented that IL-1β exposure elicited a significant enhancement in IL-6 and TNF-α levels in chondrocytes, which was partly relieved by the introduction of si-circ_0037658 (Figure 2E). Moreover, CCK-8 assay and EdU assay exhibited that IL-1β treatment might significantly inhibit cell proliferative ability, while the silence of circ_0037658 abrogated these effects (Figures 2F,G). In parallel, circ_0037658 downregulation strikingly attenuated the promotion of IL-1β on cell apoptosis (Figures 2H,I), as evidenced by decreased Bax and Cleaved-caspase-3 (Figure 2J). In terms of ECM degradation, IL-1β-mediated increase in MMP13 and decrease in Aggrecan were ameliorated by circ_0037658 deficiency in chondrocytes (Figure 2K). Collectively, circ_0037658’s absence might partly reverse IL-1β-induced chondrocyte injury.

### miR-665 Acted as a Target of Circ_0037658

**FIGURE 3:**
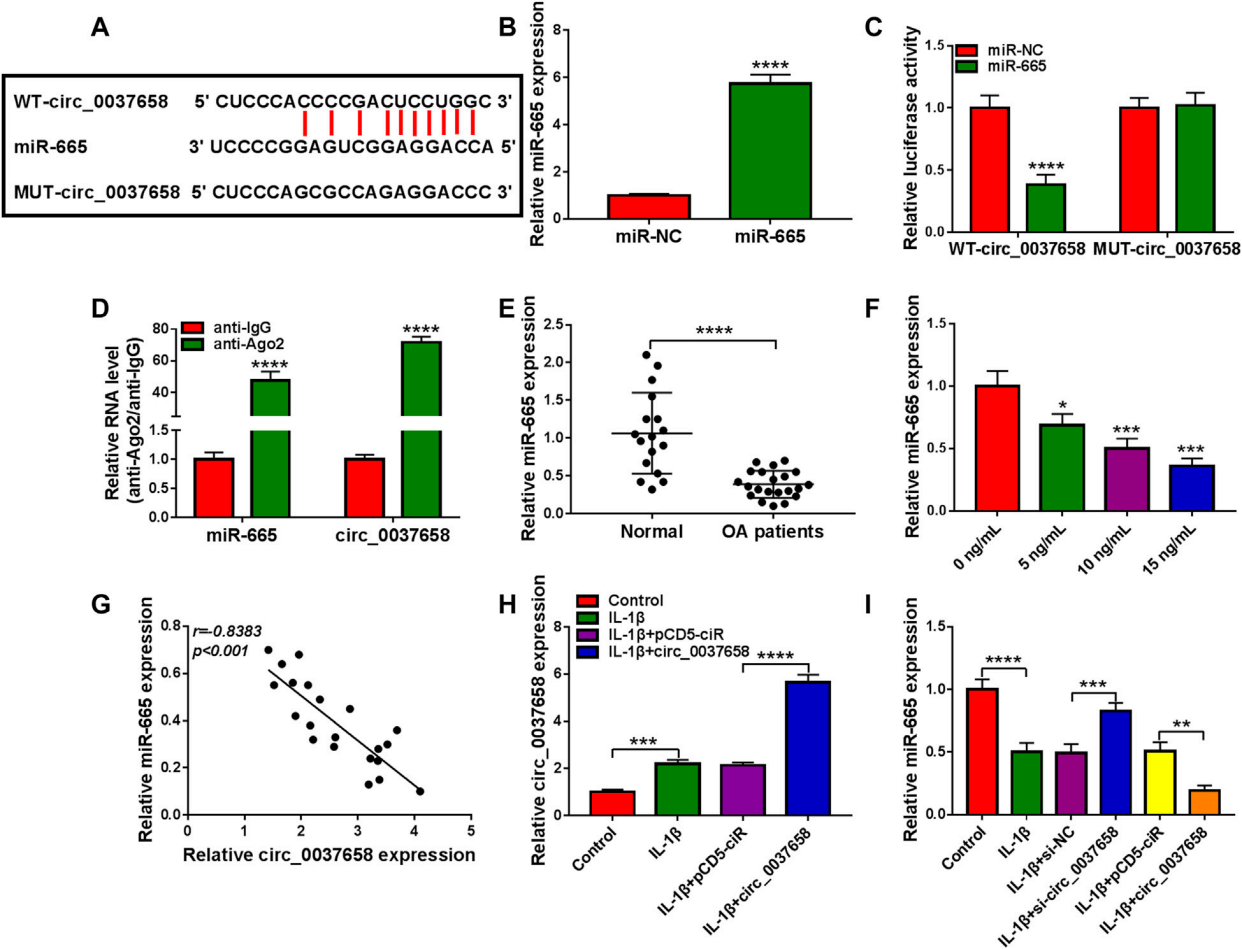
Circ_0037658 functioned as an efficient miR-665 sponge. **(A)** The predicted binding sites between circ_0037658 and miR-665. **(B)** RT-qPCR analysis of miR-665 expression (**** indicates *p* < 0.0001 relative to the miR-NC group). **(C)** A dual-luciferase reporter assay was utilized to assess binding (**** indicates *p* < 0.0001 relative to the miR-NC group). **(D)** RIP assay was performed in CHON-001 cell extracts to analyze miR-665 endogenously correlated with circ_0037658 (**** indicates *p* < 0.0001 relative to the anti-IgG group). **(E)** miR-665 level was measured in 21 OA cartilage tissues and 17 healthy cartilage tissues (**** indicates *p* < 0.0001 relative to the control group). **(F)** IL-1β (0, 5, 10 or 15 ng/ml IL-1β) on miR-665 expression was determined using RT-qPCR assay (**, ***or **** indicates *p* < 0.01, *p* < 0.001, or *p* < 0.0001 relative to the 0 ng/ml IL-1β group). **(G)** Pearson correlation analysis was utilized to analyze the expression correlation between circ_0037658 and miR-665 in OA cartilage tissues. **(H)** RT-qPCR analysis of circ_0037658 in CHON-001 cells treated with IL-1β, IL-1β+pCD5-ciR, IL-1β+circ_0037658, or without (Control) (*** indicates *p* < 0.001 when IL-1β group was compared to the negative control group and **** indicates *p* < 0.0001 when IL-1β+circ_0037658 group was compared to the IL-1β+pCD5-ciR group). **(I)** miR-665 level was determined using RT-qPCR assay in CHON-001 cells treated with IL-1β, IL-1β+si-NC, IL-1β+si-circ_0037658, IL-1β+pCD5-ciR, IL-1β+circ_0037658, or without (Control) (**** indicates *p* < 0.0001 when IL-1β group was compared to the negative control group and *** indicates *p* < 0.001 when IL-1β+si-circ_0037658 group was compared to the IL-1β+si-NC group and ** indicates *p* < 0.01 when IL-1β+circ_0037658 group was compared to the IL-1β+pCD5-ciR group). These results were presented as the mean ± (SD), *n* = 3.

To further understand the potential mechanism of circ_0037658 regulating OA development, we further searched for circ_0037658-interacting miRNAs using the online software Starbase. Data suggested that five potential target miRNAs of circ_0037658 from Starbase were found. Then, all these mRNAs in CHON-001 cells were subjected to RT-qPCR analysis responding to circ_0037658 downregulation. Among these five miRNAs, miR-665 displayed the highest fold change (Supplementary Figure S1). Therefore, we chose miR-665 for further research. Furthermore, some binding sites between circ_0037658 and miR-665 were exhibited in Figure 3A. Meanwhile, miR-665 expression was significantly improved in miR-665 mimic-transfected cells (Figure 3B). Then, data presented only combined miR-665 mimic and WT-circ_0037658 transfection reduced luciferase activity in chondrocytes (Figure 3C). Synchronously, circ_0037658 and miR-665 could be abundantly precipitated together by the Ago2 antibody compared with the IgG antibody using RIP assay (Figure 3D), further verifying the combination between circ_0037658 and miR-665. Of interest, the RT-qPCR assay presented a significant decrease of miR-665 in 21 OA cartilage tissues compared with 17 healthy cartilage tissues (Figure 3E). Moreover, miR-665 content was gradually reduced in IL-1β-treated CHON-001 cells (Figure 3F). According to Pearson correlation analysis, circ_0037658 was negatively associated with miR-665 in OA cartilage tissue samples (Figure 3G). Moreover, the RT-qPCR assay displayed that the circ_0037658 level was significantly increased in IL-1β-triggered CHON-001 cells after the transfection of circ_0037658 (Figure 3H), suggesting that the pCD5-circ_0037658 overexpressing vector was successfully transfected into IL-1β-administered CHON-001 cells. Then, the introduction of si-circ_0037658 enhanced the miR-665 level in IL-1β-induced CHON-001 cells, and pCD5-circ_0037658 exhibited the opposite results (Figure 3I). Overall, circ_0037658 interacted with miR-665.

### Circ_0037658 Downregulation Might Mitigate IL-1β-Evoked Chondrocyte Injury by Targeting miR-665

**FIGURE 4:**
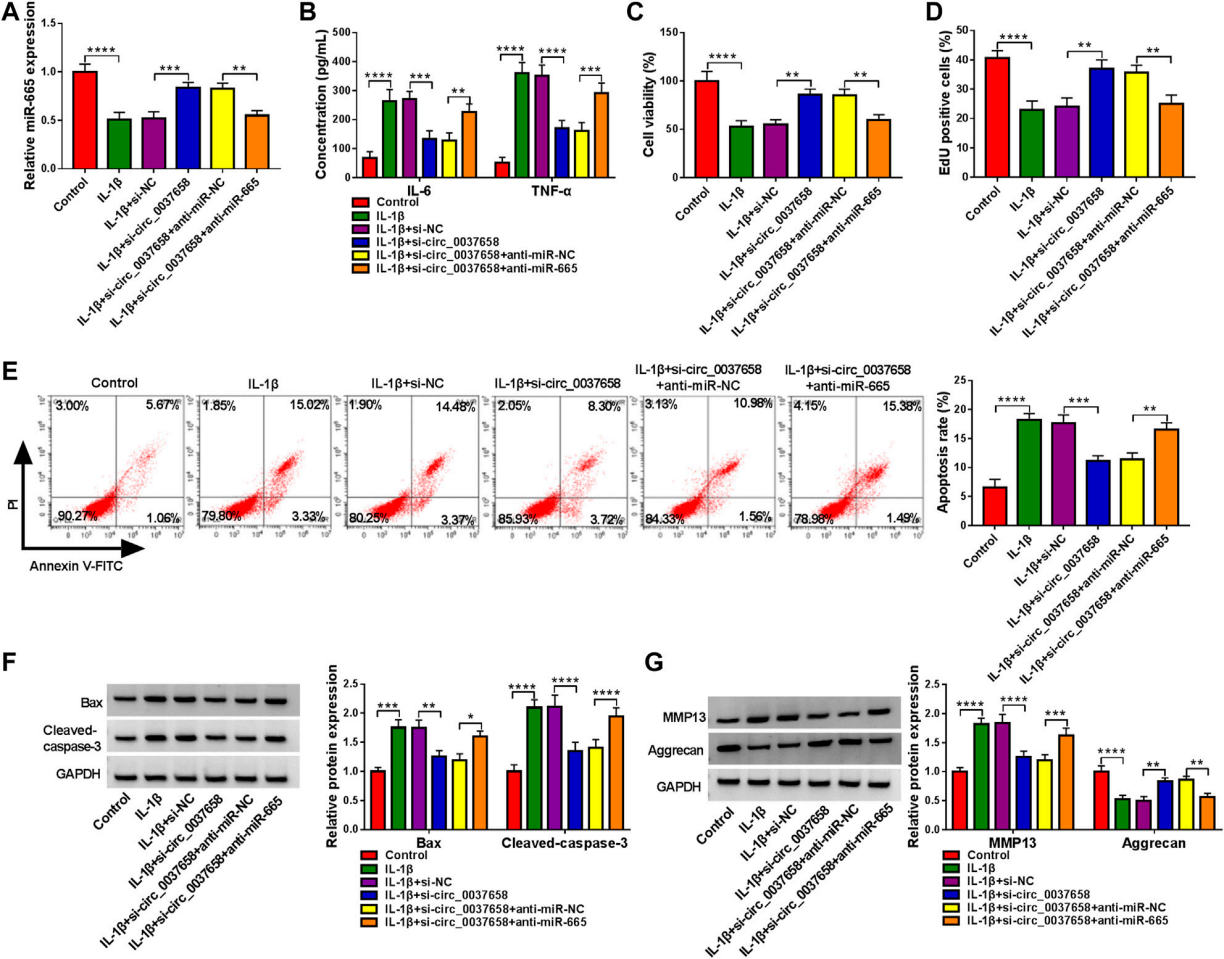
Downregulation of miR-665 counteracted the effect of circ_0037658 on inflammation, proliferation, apoptosis, and ECM degradation in IL-1β-treated chondrocytes. CHON-001 cells were treated with IL-1β, IL-1β+si-NC, IL-1β+si-circ_0037658, IL-1β+si-circ_0037658+miR-NC, IL-1β+si-circ_0037658+miR-665, or without (Control). **(A)** miR-665 level was detected in treated CHON-001 cells using RT-qPCR assay. **(B)** ELISA was adopted for IL-6 and TNF-α level in treated CHON-001 cells. **(C,D)** CCK-8 assay and EdU assay were applied to analyze cell proliferative ability in treated CHON-001 cells. **(E)** Cell apoptosis rate in treated CHON-001 cells. **(F,G)** Western blot analysis was utilized for Bax, Cleaved-caspase-3, MMP13, and Aggrecan protein levels in each group, and GAPDH was used as a loading control. **** or *** indicates *p* < 0.0001 or *p* < 0.001 when IL-1β group was compared to the negative control group and **, ***, or **** indicates *p* < 0.01, *p* < 0.001 or *p* < 0.0001 when IL-1β+si-circ_0037658 group was compared to the IL-1β+si-NC group and **, ***, or **** indicates *p* < 0.01, *p* < 0.001 or *p* < 0.0001 when IL-1β+si-circ_0037658+anti-miR-665 group was compared to the IL-1β+si-circ_0037658+anti-miR-NC group. These results were presented as the mean ± (SD), *n* = 3.

Then, we further explored the influence of circ_0037658 and miR-665 on IL-1β-induced cell damage. After diverse transfection, we found that the co-transfection of anti-miR-665 might significantly counteract circ_0037658 downregulation-induced miR-665 level promotion in IL-1β-treated CHON-001 cells (Figure 4A). Functionally, circ_0037658 silencing might significantly block the secretion of these pro-inflammatory cytokines in the treated CHON-001 cells, while miR-665 downregulation could effectively abolish these effects (Figure 4B). In contrast, the miR-665 inhibition partly overturned the positive impact of circ_0037658 silencing on cell proliferative ability in IL-1β-exposed CHON-001 cells (Figures 4C,D). In addition, circ_0037658 silencing repressed CHON-001 cell apoptosis rate, but anti-miR-665 ameliorated these effects in treated cells (Figure 4E), accompanied by higher Bax and Cleaved-caspase-3 levels (Figure 4F). Furthermore, the si-circ_0037658-mediated MMP13 reduction and Aggrecan increase were attenuated by miR-665 inhibitor in IL-1β-treated CHON-001 cells, meaning that miR-665 knockdown might abolish the repression effect of circ_0037658 deletion on ECM degradation of CHON-001 cells (Figure 4G). All in all, miR-665’s absence partly weakened the suppressive role of si-circ_0037658 on IL-1β-triggered CHON-001 cell injury.

### ADAMTS5 Acted as a Target of miR-665

**FIGURE 5:**
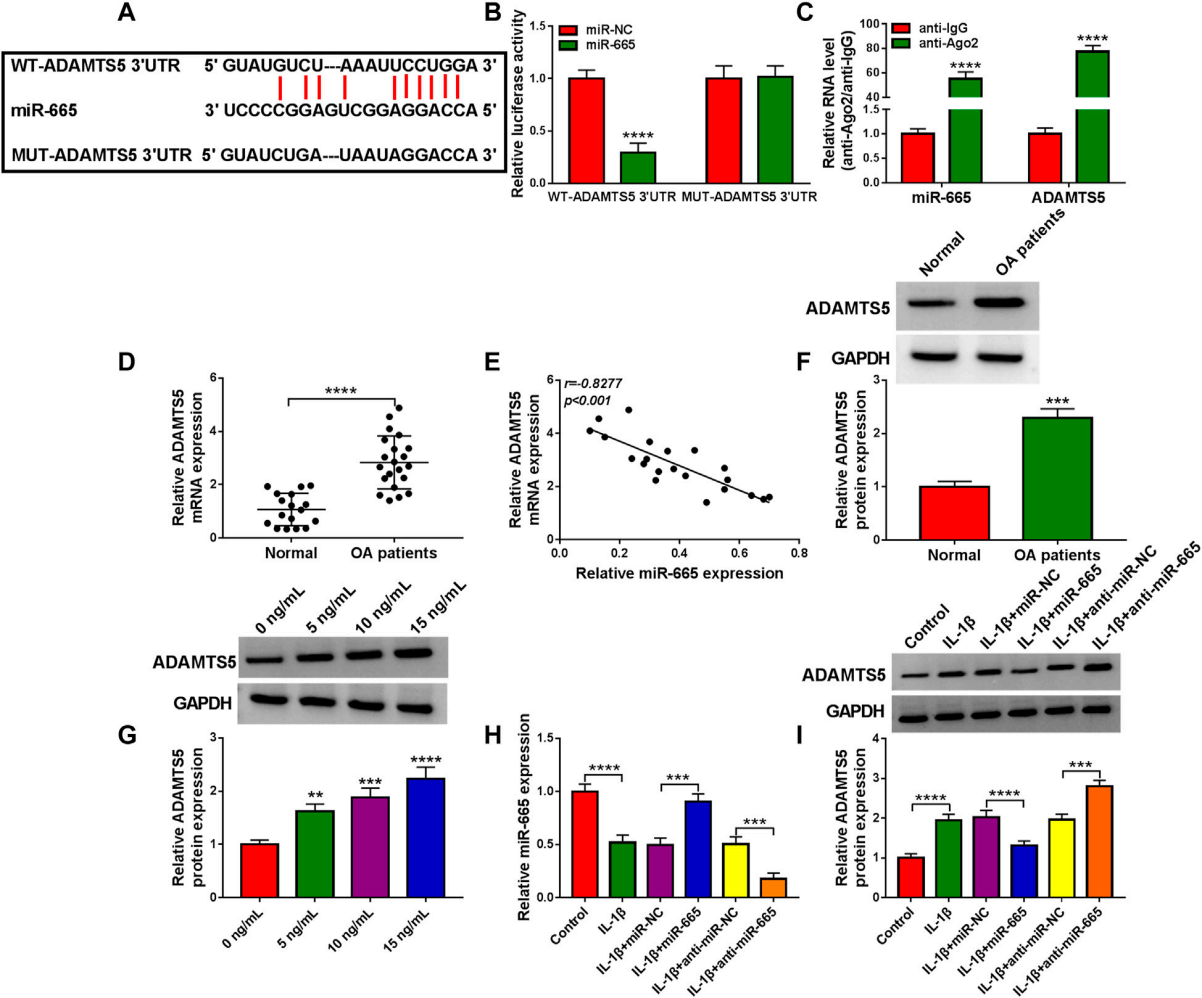
miR-665 is directly targeted by ADAMTS5. **(A)** Binding between ADAMTS5 and miR-665 was analyzed using Starbase software. **(B,C)** The binding was validated using a dual-luciferase reporter assay and RIP assay in CHON-001 cells (**** indicates *p* < 0.0001 relative to the miR-NC group or the anti-IgG group). **(D)** ADAMTS5 expression was determined in 21 OA cartilage tissues and 17 healthy cartilage tissues using RT-qPCR assay (**** indicates *p* < 0.0001 relative to the control group). **(E)** The expression correlation between miR-665 and ADAMTS5 in OA cartilage tissues was analyzed by Pearson correlation analysis. **(F)** ADAMTS5 protein level was measured in 21 OA cartilage tissues and 17 healthy cartilage tissues using western blot assay (GAPDH was used as a loading control) (*** indicates *p* < 0.001 relative to the control group). **(G)** Western blot analysis of ADAMTS5 protein level in CHON-001 cells treated with various doses of IL-1β, and GAPDH was used as a loading control (**, ***or **** indicates *p* < 0.01, *p* < 0.001, or *p* < 0.0001 relative to the 0 ng/ml IL-1β groups). **(H,I)** CHON-001 cells were treated with IL-1β, IL-1β+ miR-NC, IL-1β+miR-665, IL-1β+anti-miR-NC, IL-1β+anti-miR-665, or without (Control), then, miR-665 level was assessed using RT-qPCR assay and ADAMTS5 protein level was measured using western blot assay (**** indicates *p* < 0.001 when IL-1β group was compared to the negative control group and *** or **** indicates *p* < 0.001 or *p* < 0.0001 when IL-1β+miR-665 group was compared to the IL-1β+ miR-NC group and *** indicates *p* < 0.001 when IL-1β+anti-miR-665 group was compared to the IL-1β+anti-miR-NC group). These results were presented as the mean ± (SD), *n* = 3.

A predicted miR-665-binding site was found in the 3′UTR of ADAMTS5 (Figure 5A). Then, miR-665 overexpression significantly decreased the luciferase activity of the WT-ADAMTS5 3′UTR reporter vector but not that of the MUT-ADAMTS5 3′UTR reporter vector in CHON-001 cells (Figure 5B). Consistently, miR-665 and ADAMTS5 levels were enriched in the Ago2 immunoprecipitates using the RIP assay (Figure 5C). Interestingly, inversely associated with miR-665 expression (Figure 5E), ADAMTS5 was raised in OA cartilage tissues (Figures 5D,F). As we expected, the ADAMTS5 protein level was gradually enhanced in CHON-001 cells after the treatment of 0–15 ng/ml IL-1β (Figure 5G). Furthermore, the overexpression or knockdown efficiency of miR-665 in CHON-001 cells was measured using RT-qPCR assay (Figure 5H). Then, the western blot assay displayed that miR-665 upregulation significantly reduced ADAMTS5 expression in CHON-001 cells, whereas the miR-665 inhibitor presented an opposite result (Figure 5I). Overall, ADAMTS5 acts as a direct downstream target gene of miR-665.

### ADAMTS5 Overexpression Reversed the Effects of miR-665 on Inflammation, Proliferation, Apoptosis, and ECM Degradation in IL-1β-Treated Chondrocytes

**FIGURE 6:**
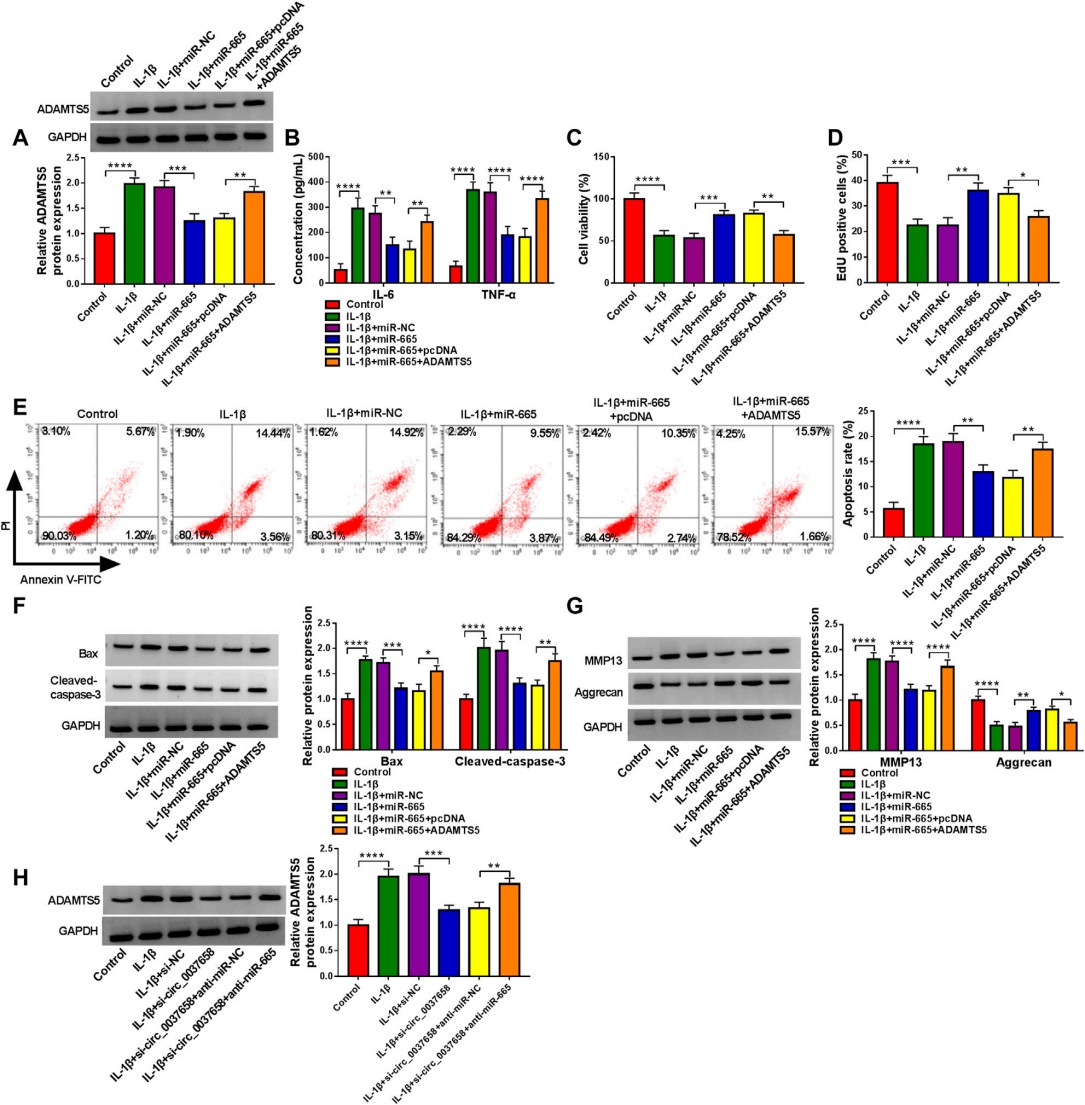
miR-665-mediated inflammation, proliferation, apoptosis, and ECM degradation was abolished by interacting with ADAMTS5. **(A–G)** CHON-001 cells were treated with IL-1β, IL-1β+ miR-NC, IL-1β+miR-665, IL-1β+miR-665 + pcDNA, IL-1β+miR-665 + ADAMTS5, or without (Control). **(A)** ADAMTS5 protein level was determined in treated CHON-001 cells using western blot assay. **(B)** IL-6 and TNF-α levels in treated CHON-001 cells were examined by ELISA kit. **(C,D)** CHON-001 cell proliferative ability was measured by CCK-8 assay and EdU assay. **(E)** CHON-001 cell apoptosis rate was monitored by flow cytometry assay. **(F,G)** Protein levels of Bax, Cleaved-caspase-3, MMP13, and Aggrecan were determined by western blot assay. **(H)** ADAMTS5 protein level was detected in CHON-001 cells were treated with Control, IL-1β, IL-1β+si-NC, IL-1β+si-circ_0037658, IL-1β+si-circ_0037658+anti-miR-NC, and IL-1β+si-circ_0037658+anti-miR-665 by western blot assay. **** or *** indicates *p* < 0.0001 or *p* < 0.001 when IL-1β group was compared to the negative control group and **, ***, or **** indicates *p* < 0.01, *p* < 0.001 or *p* < 0.0001 when IL-1β+miR-665 group was compared to the IL-1β+ miR-NC group and *, **, or **** indicates *p* < 0.05, *p* < 0.01, or *p* < 0.0001 when IL-1β+miR-665 + ADAMTS5 was compared to IL-1β+miR-665 + pcDNA. These results were presented as the mean ± (SD), *n* = 3.

Then, to elucidate their correlation cell model of OA, we conducted rescue assays. As displayed in Figure 6A, miR-665 overexpression significantly declined ADAMTS5 protein level in CHON-001 cells, while these effects were partly counteracted after co-transfection with pcDNA-ADAMTS5. Subsequently, ELISA exhibited that ADAMTS5 upregulation might overturn miR-665 mimic-mediated IL-6 and TNF-α levels inhibition in IL-1β-induced CHON-001 cells (Figure 6B). Meanwhile, the overexpression of miR-665 significantly elevated cell proliferative ability, whereas pcDNA-ADAMTS5 partly reversed this impact in CHON-001 cells (Figures 6C,D). Simultaneously, the upregulation of ADAMTS5 in IL-1β-stimulated CHON-001 cells effectively ameliorated the repression of miR-665 overexpression on cell apoptosis rate (Figure 6E), which was further confirmed by Bax and Cleaved-caspase-3 expression (Figure 6F). For ECM degradation, the re-introduction of pcDNA-ADAMTS5 might partly relieve the miR-665-triggered decline in MMP13 and increase in Aggrecan in CHON-001 cells (Figure 6G). Then, western blot indicated that the deficiency of circ_0037658 might dampen ADAMTS5 protein expression in CHON-001 cells, and anti-miR-665 might significantly attenuate the impact (Figure 6H), suggesting the circ_0037658/miR-665/ADAMTS5 axis. In summary, miR-665 might abate IL-1β-triggered cell damage by targeting ADAMTS5.

## Discussion

Recently, with the development of bioinformatics analysis and high-throughput sequencing technology, a larger number of circRNAs have been identified in mammalian cells (López-Jiménez et al., 2018; Sekar et al., 2019). Unlike linear RNAs, most of them presented are highly stable and abundantly expressed in extracellular fluid, emphasizing their significance for diagnostic molecular biomarkers in diverse diseases, containing OA (Yu and Sun, 2018). Some scholars discovered that plentiful circRNAs participate in many pathological processes during OA (Shen et al., 2019; Mao et al., 2021). Previous studies have indicated that synovial fibroblasts (SFB) may influence the formation of osteophytes and the degradation of the cartilage matrix by releasing pro-inflammatory factors (such as IL-1β) in an inflammatory environment (Mathiessen and Conaghan, 2017). Therefore, IL-1β is a crucial inflammatory factor, which has been confirmed to be linked to OA etiology-related cartilage degeneration (Wojdasiewicz et al., 2014). At present, *in vitro,* it has been widely accepted that IL-1β-treated chondrocytes were used to construct an OA pathological model*.* In this regard, the current work exhibited the IL-1β-treated OA cell model. Notably, our data displayed that circ_0037658 was a typical circular RNA, which was exceptionally increased in IL-1β-stimulated CHON-001 cells and OA patients’ cartilage tissue, in accordance with a previous report (Sui et al., 2021), implying that circ_0037658 might perform as an attractive biomarker in OA research.

Previous studies have discovered that phenotypic stability and chondrocyte survival are critical for maintaining an appropriate cartilage matrix (Singh et al., 2019; Trachana et al., 2019). That is, chondrocyte dysfunction is now considered to be a vital contributor to the pathogenesis of OA. Inflammation is known as one of the hallmarks of OA, which can disturb cellular energy balance and enhance cell stress (Liu-Bryan, 2015; Mobasheri et al., 2015). In this paper, circ_0037658 knockdown blocked an IL-1β-induced inflammatory response in CHON-001 cells. Meanwhile, a central feature in OA progression was recognized as abnormal chondrocyte proliferation and apoptosis (Hwang and Kim, 2015). The present research suggests that IL-1β-mediated reduced proliferation and improved apoptosis were abolished via circ_0037658 downregulation. Additionally, ECM degradation underlies the loss of cartilage tissue in OA (Shi et al., 2019). In the review, IL-1β treatment elicited an increase in MMP13 (ECM-degrading enzyme) and a decline in Aggrecan (the main component of ECM) in CHON-001 cells, which were overturned after introduction with si-circ_0037658. These findings supported that circ_0037658 knockdown could attenuate IL-1β-evoked chondrocyte injury *in vitro*.

Several studies have stated a hypothesis of competitive endogenous RNAs (ceRNAs), suggesting that circRNAs can be competitively bound to common miRNA response elements for mutual regulation (Hansen et al., 2013; Panda, 2018). However, the association between circ_0037658 and miRNA seemed to be lacking in previous work. In this paper, miR-665 was of particular interest among putative target miRNAs of circ_0037658. MiR-665 has been confirmed to play an important role in the circ_RUNX2-miRNA’s regulatory network in OA progression (Wang C. et al., 2021). Furthermore, the dysregulation of miR-665 was closely associated with IL-1β-triggered cell damage in some relevant literature (Wang et al., 2020; Ouyang et al., 2021). The current work highlights that circ_0037658 directly targets miR-665. As expected, low miR-665 expression overturned the effects of circ_0037658 silencing, thereby recovering IL-1β-triggered cell damage *in vitro*. That was, circ_0037658 deficiency weakened the IL-1β-induced chondrocyte injuries by acting as a molecular sponge of miR-665. Analogously, using online software, ADAMTS5 was deemed as the potential downstream target mRNA of miR-665 in this research. It has been verified that ADAMTS5 is a major cartilage degrading enzyme in arthritis and is positively related to articular cartilage degradation (McCulloch et al., 2009; Ji et al., 2016). Synchronously, a prior report validated that ADAMTS5 takes part in the regulation of IL-1β-evoked chondrocyte viability and cartilage matrix degradation in OA (Liu et al., 2019). Consistent with the former paper (Lu et al., 2016), ADAMTS5 was identified to be highly expressed in OA patients and raised by IL-1β.Meanwhile, our results confirmed that enhanced ADAMTS5 might partly relieve the suppressive role of miR-665 on IL-1β-evoked cell damage. Intriguingly, our results verified that circ_0037658 silencing might constrain ADAMTS5 expression in IL-1β-treated chondrocytes, and the re-introduction of miR-665 inhibitor partially abolished these effects. These results further supported the circ_0037658-miR-665-ADAMTS5 axis in IL-1β-treated chondrocytes. Frankly speaking, there are several limitations to this research. For example, the function of circ_0037658 was mainly explored in the OA cell models, but animal experiments were still lacking, and more clinical assays need to be conducted in the future.

## Conclusion

Taken together, these results delineated the first evidence that circ_0037658 could regulate IL-1β-triggered chondrocyte injury in part by targeting the miR-665/ADAMTS5 axis, implying a vital preclinical basis for OA treatment.

## Data Availability

The original contributions presented in the study are included in the article/Supplementary Material; further inquiries can be directed to the corresponding author.

## Supplementary Material

Supplementary Figure S1 miR-665 expression was increased in si-circ_0037658-treated CHON-001 cells. RT-qPCR analysis of miR-107, miR-15a-5p, miR-424-5p, miR-296-3p, and miR-665 expression in CHON-001 cells transfected with si-NC or si-circ_0037658. ** or **** indicates *P* <0.01 or *P* <0.0001 relative to the si-NC groups. These results were presented as the mean ± (SD), *n*=3.
