# Supplementary material for: Knockdown of Circ_0037658 Alleviates IL-1β-Induced Osteoarthritis Progression by Serving as a Sponge of miR-665 to Regulate ADAMTS5
Source: Front Genet. 2022 Aug 24;13:886898. doi: 10.3389/fgene.2022.886898 (PMC9449488; doi:10.3389/fgene.2022.886898)
Supplement: Supplementary file 1 [file Table1.DOC]

Table 1 The sequences of primers for RT-qPCR used in this study

| Names | Sequences (5’-3’) |
| --- | --- |
| hsa_circ_0037658: Forward | CCAGACGACAATTTCAAAGGA |
| hsa_circ_0037658: Reverse | GGTGCAGTGGTGACTGTGTC |
| ADAMTS5: Reverse | AAAGGGGAGAATCTGCCTGC |
| ADAMTS5: Reverse | CCAAGATCCCCAGTTGCCAT |
| miR-665: Forward | GTATGAGACCAGGAGGCTGA |
| miR-665: Reverse | CTCAACTGGTGTCGTGGAG |
| U6: Forward | CTCGCTTCGGCAGCACATA |
| U6: Reverse | CGAATTTGCGTGTCATCCT |
| GAPDH: Forward | AAGGCTGTGGGCAAGGTCATC |
| GAPDH: Reverse | GCGTCAAAGGTGGAGGAGTGG |
|  |  |
