# Supplementary figures and images for: Knockdown of Circ_0037658 Alleviates IL-1β-Induced Osteoarthritis Progression by Serving as a Sponge of miR-665 to Regulate ADAMTS5
Source: Front Genet. 2022 Aug 24;13:886898. doi: 10.3389/fgene.2022.886898 (PMC9449488; doi:10.3389/fgene.2022.886898)

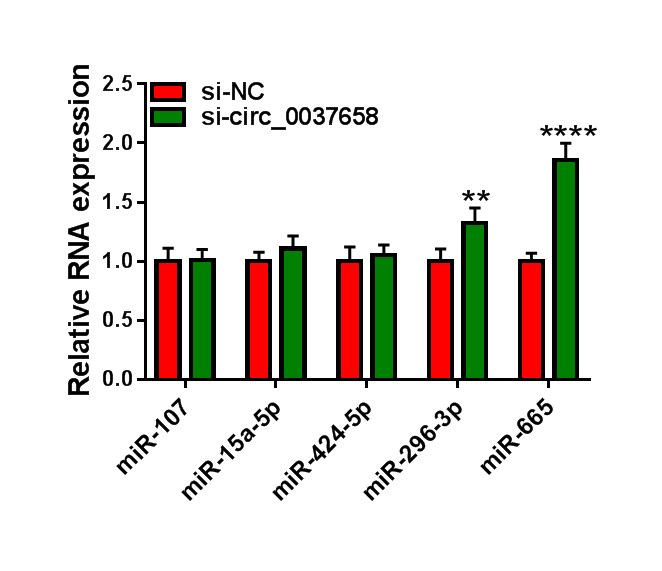

Supplement: Supplementary file 2 [file Figure7.TIF]
